# Widespread Distribution of a Newly Found Point Mutation in Voltage-Gated Sodium Channel in Pyrethroid-Resistant *Aedes aegypti* Populations in Vietnam

**DOI:** 10.1371/journal.pntd.0000527

**Published:** 2009-10-06

**Authors:** Hitoshi Kawada, Yukiko Higa, Osamu Komagata, Shinji Kasai, Takashi Tomita, Nguyen Thi Yen, Luu Lee Loan, Rodrigo A. P. Sánchez, Masahiro Takagi

**Affiliations:** 1 Department of Vector Ecology & Environment, Institute of Tropical Medicine, Nagasaki University, Nagasaki, Japan; 2 National Institute of Infectious Diseases, Tokyo, Japan; 3 National Institute of Hygiene and Epidemiology, Hanoi, Vietnam; 4 Pasteur Institute, Ho Chi Minh City, Vietnam; 5 Universidad Evangélica de El Salvador, San Salvador, El Salvador; Yale School of Public Health, United States of America

## Abstract

**Background:**

Resistance of *Aedes aegypti* to photostable pyrethroid insecticides is a major problem for disease-vector control programs. Pyrethroids target the voltage-gated sodium channel on the insects' neurons. Single amino acid substitutions in this channel associated with pyrethroid resistance are one of the main factors that cause knockdown resistance in insects. Although *kdr* has been observed in several mosquito species, point mutations in the para gene have not been fully characterized in *Ae. aegypti* populations in Vietnam. The aim of this study was to determine the types and frequencies of mutations in the para gene in *Ae. aegypti* collected from used tires in Vietnam.

**Methods and Findings:**

Several point mutations were examined that cause insensitivity of the voltage-gated sodium channel in the insect nervous system due to the replacement of the amino acids L1014F, the most commonly found point mutation in several mosquitoes; I1011M (or V) and V1016G (or I), which have been reported to be associated to knockdown resistance in *Ae. aegypti* located in segment 6, domain II; and a recently found amino acid replacement in F1269 in *Ae. aegypti*, located in segment 6, domain III. Among 756 larvae from 70 locations, no I1011M or I1011V nor L1014F mutations were found, and only two heterozygous V1016G mosquitoes were detected. However, F1269C mutations on domain III were distributed widely and with high frequency in 269 individuals among 757 larvae (53 collection sites among 70 locations surveyed). F1269C frequencies were low in the middle to north part of Vietnam but were high in the areas neighboring big cities and in the south of Vietnam, with the exception of the southern mountainous areas located at an elevation of 500–1000 m.

**Conclusions:**

The overall percentage of homozygous F1269C seems to remain low (7.4%) in the present situation. However, extensive and uncontrolled frequent use of photostable pyrethroids might be a strong selection pressure for this mutation to cause serious problems in the control of dengue fever in Vietnam.

## Author Summary

Pyrethroid is the general term for a group of synthetic chemicals that are structurally related to natural pyrethrins derived from C*hrysanthemum* flowers. In Vietnam, photostable pyrethroids have been extensively used as insecticides for malaria and dengue vector control. Recently, Kawada et al. found that *Aedes aegypti* collected from used tires were susceptible to pyrethroids in the North but were resistant in the South and Central Highlands of Vietnam. By analyzing the presence of mutations in the para locus, which cause insensitivity to voltage-gated sodium channel in the insect nervous system, a recently found amino acid replacement in the area of segment 6 of domain III (F1269C) was found to be widely distributed with high frequency in southern Vietnam. We suggest that this point mutation plays an important role in pyrethroid resistance in Vietnam. Extensive and uncontrolled frequent use of photostable pyrethroids might be a strong selection pressure for this mutation to increase homozygous populations, which cause serious problems in controlling dengue fever in Vietnam.

## Introduction

Pyrethroid is the general term for a group of synthetic chemicals that are structurally related to natural pyrethrins derived from C*hrysanthemum* flowers. There are two main groups of pyrethroids: One possessing high knockdown activity but low killing activity and the other possessing high killing activity. Pyrethroids in the former group such as *d*-allethrin, prallethrin, metofluthrin etc. are labeled as knockdown agents, and those in the latter group such as permethrin, deltamethrin, cypermethrin etc., as killing agents. The pyrethroids belonging to the former group generally exhibit low stability in the environment. Pyrethroids are typically used as a ‘spatial repellent’ in mosquito coils, mats, and vaporizer liquids to prevent mosquito bites. The use of such pyrethroids is believed to be biorational since it does not kill the affected insects, it causes no selection pressure on insect populations, and mosquitoes develop minimum physiological resistance. The pyrethroids belonging to the latter group, on the other hand, generally exhibit high photostability that enables their outdoor use. Due to their high killing activity, photostable pyrethroids are emerging as the predominant insecticides for vector control. In fact, globally, photostable pyrethroids comprise 40% of the insecticides used annually for indoor residual spraying against malaria vectors and 100% of the World Health Organization (WHO)-recommended insecticides for the treatment of mosquito nets [1]. In Vietnam, photostable pyrethroids have been extensively used in large amounts for malaria and dengue vector control after abandonment of DDT [1]–[4]. Recently, Kawada et al. reported the widespread distribution of pyrethroid resistance in *Aedes aegypti* (L.) in southern Vietnam. They also suggested a correlation between pyrethroid resistance and the total annual pyrethroid use for malaria vector control (1998–2007) [3]. Vu et al. and Huber et al. also reported a similar tendency in pyrethroid susceptibility in *Ae. aegypti* in Vietnam [5],[6].

Resistance to photostable pyrethroids is believed to be a major problem for the vector control program. Moreover, cross-resistance to such killing agents in addition to knockdown agents is a significant concern. Two different mechanisms are involved in pyrethroid resistance: Enhanced metabolic detoxification and the insensitivity of target sites. Pyrethroids target the voltage-gated sodium channel on the insects' neurons. Single amino acid substitutions in this channel are associated with pyrethroid resistance and are known as knockdown resistance (*kdr*). These *kdr*-type resistances have been observed in several mosquitoes, such as *Anopheles gambiae* Giles [7], *Anopheles stephensi* Liston [8], *Culex quinquefasciatus* Say [9], and *Ae. aegypti* [10]. In *Culex* and *Anopheles*, *kdr* resistance is associated with the replacement of a leucine at position 1014 in segment 6 of domain II with either phenylalanine or serine [7]–[9]. Brengues et al. described several mutations in segment 6 of domain II of para in *Ae. aegypti* [10], and Saavedra-Rodriguez et al. found additional mutations at the same position (I1011M, I1011V, V1016G, and V1016I) [11]. Chang et al. found a novel mutation D1794Y, located within the extracellular linker between segment 5 and segment 6 of domain IV, which is concurrent with the known V1023G (this is identical to the above mentioned V1016G by the notational system used in the present paper) mutation in permethrin-resistant *Ae. aegypti* [12]. Recently, Yanola et al. found a novel mutation, F1269C, in segment 6 of domain III in DDT/permethrin-resistant *Ae. aegypti* [13]. However, no such genetic analyses of point mutations in the voltage-gated sodium channel have been performed on *Ae. aegypti* colonies in Vietnam except for one colony (Long Hoa strain) [8]. The objective, then, of the present study was to elucidate the mechanisms of pyrethroid resistance in *Ae. aegypti* collected from used tires in Vietnam [3] by analyzing the presence of mutations. Our targets were the three most frequent amino acid replacements in I1011, L1014, and V1016, all of which are located in the area of segment 6 of domain II, and a recently found amino acid replacement at F1269 located in the area of segment 6 of domain III [13].

## Materials and Methods

### Collection of *Ae. aegypti* larvae from used tires and simplified knockdown bioassay

We periodically drove along the national road from the north end to the Mekong Delta in Vietnam (7–16 December 2006; 17–20 March, 15–20 May, and 1–12 July 2007; and 7–16 January 2008) and collected mosquito larvae from used tires, as previously described by Kawada et al. [3]. Using the larvae obtained from the collection sites, a simplified bioassay for the detection of knockdown susceptibility was carried out on the day of collection [3]. Each larva was placed in a glass vial with 20 ml of water. An emulsifiable concentrate of 90% *d*-T_80_-allethrin was diluted with water to obtain a 250-ppm solution. After releasing the larva, 32 or 8 µl of the solution was added in each vial to obtain a concentration of 0.4 or 0.1 ppm, respectively. Twenty larvae from each site were used for each concentration regime. Larval knockdown was observed for 30 min. Larvae that sank to the bottom of the glass vial and could not swim, float, or were paralyzed were judged as knocked down larvae, and the time to knockdown was recorded for each larva. The median knockdown times (KT_50_s), i.e., the time required for 50% knockdown, were scored according to the 6 following categories: 1, <5 min; 2, 5–10 min; 3, 10–15 min; 4, 15–20 min; 5, 20–30 min; and 6, >30 min. The susceptibility index was calculated as the product of the scores at 0.1 and 0.4 ppm. Thus, mosquitoes with a susceptibility index of 1 were considered to be the most susceptible, and those with a susceptibility index of 36 were considered to be the least susceptible to *d*-allethrin. After the bioassay, each larva was stored in a 1.5-ml plastic vial containing ethanol solution for subsequent polymerase chain reaction (PCR) analysis.

### Analysis of frequency of the point mutations

In order to verify the presence of the point mutations at I1011, L1014, V1016, and F1269 in *Ae. aegypti*, PCR and direct DNA sequencing was done. After bioassay, 12 larvae from each collection site were randomly selected for the analysis. The mosquito sample (a larva immersed in ethanol) was lightly dried on a paper towel and then placed in a 1.5-ml PCR reaction tube. The sample was homogenized in a mixed solution of extraction solution (40 µl)+tissue-preparation solution (10 µl) (REDExtract-N-Amp™ Tissue PCR Kit; SIGMA, St. Louis, MO, USA) for extraction of DNA. The solution was heated at 95°C for 3 min and was neutralized. Initial fragment amplification was carried out using primers AaSCF1 (AGACAATGTGGATCGCTTCC) and AaSCR4 (GGACGCAATCTGGCTTGTTA) for I1011M (or (V), L1014F, and V1016G (or I) analysis, or AaSCF7 (GAGAACTCGCCGATGAACTT) and AaSCR7 (GACGACGAAATCGAACAGGT) for F1269 analysis, respectively. The PCR mixture contained 4 µl of REDExtract-N-Amp™ ReadyMix (SIGMA), 0.5 µM of each primer, and 1 µl of the DNA template in a total volume of 10 µl. PCR was performed under the following conditions: 94°C for 3 min and 35 cycles of 94°C for 15 s, 55°C for 30 s, 72°C for 30 s; and 72°C for 10 min. The amplified fragments of the expected size were purified using ExoSAP-IT (USB Corporation, Cleveland, OH, USA) at a temperature of 37°C for 30 min and then 80°C for 15 min. DNA sequencing was carried out using primers AaSCF3 (GTGGAACTTCACCGACTTCA) and AaSCR6 (CGACTTGATCCAGTTGGAGA) for I1011M (or V), L1014F, and V1016G (or I) analysis or AaSCR8 (TAGCTTTCAGCGGCTTCTTC) for F1269C analysis, respectively. A BigDye Terminator v 3.1 Cycle Sequencing Kit (Applied Biosystems Japan Ltd., Tokyo, Japan) was used for DNA sequencing according to the manufacturer's instructions. Two micromoles of each primer were added to a tube with a total mixture volume of 10 µl. PCR was performed under the following conditions: 96°C for 1 min and 25 cycles of 96°C for 10 s, 50°C for 5 s, 60°C for 2 min. Ethanol precipitation was performed by adding 1 µl each of 0.125-M EDTA solution and 3-M sodium acetate and 25 µl of 100% ethanol to a 5-µl sample of the above reacted solution. The sample was centrifugated at 4°C at 3,000×*g* for 30 min, after which the supernatant was removed, and 35 µl of 70% ethanol was added. The resulting mixture was centrifugated at 4°C at 3,000×*g* for 15 min, and the supernatant was removed. Finally, 10 µl of Hi-Di Formamide (Applied Biosystems, Foster City, CA, USA) was added to the sediment and heated at 95°C for 2 min. Direct DNA sequencing was performed using the 3730 DNA Analyzer (Applied Biosystems). The electropherogram of the targeted amino acid replacement was analyzed by MEGA 4.0 public domain software (http://www.megasoftware.net/).

### Statistical analysis

The susceptibility index and the corresponding frequency of point mutations for each collection point were plotted on a map using ArcGIS 9.3 (ESRI Japan Corp, Tokyo, Japan.). Univariate analysis was performed to analyze the correlation between the susceptibility indices and the frequencies of point mutations using JMP 7.0 (SAS Institute Inc., Cary, NC, USA).

## Results

**Figure 1:**
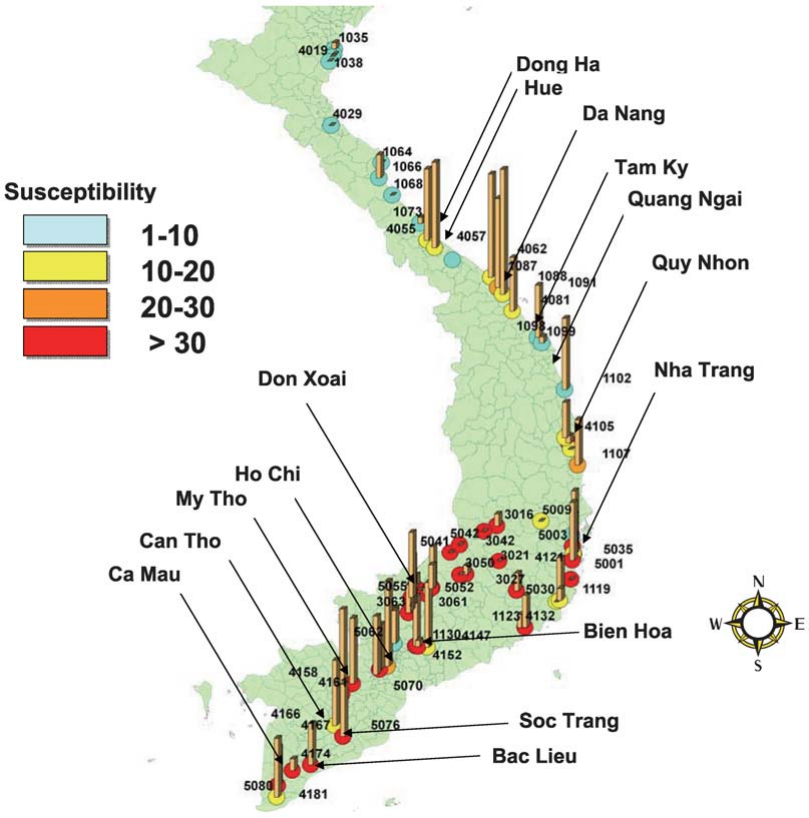
The distribution of the F1269C point mutation in *Aedes aegypti* collected from used tires in Vietnam. The date and location of the mosquito collection from used tires in Vietnam are indicated. The bars indicate relative frequency of F1269C mutations in the samples collected in one collection site. The color in each circle indicates the susceptibility index, after Kawada et al. [3].

Susceptibility indices [3], the allelic frequencies of point mutations, and the percentage of homozygous of mutations per larvae of *Ae. aegypti* collected from used tires in Vietnam are shown in Table 1 and Figure 1. Among the 706 larvae collected from 69 locations in Vietnam, no I1011M or I1011V mutation was found. Similarly, among the 735 larvae collected from 70 locations, not a single L1014F mutation was found. For the V1016G point mutation, only two heterozygous individuals among 756 larvae from 70 locations were found from one location (allelic frequency, 20%), the Binh Son district, Quang Ngai province, central east coast of Vietnam (site no. 1099). Overall allelic frequency of V1016G was 0.26%. Conversely, the recently found F1269C mutation on domain III was distributed widely and had a high allelic frequency in 269 individuals among 757 larvae (35.5%). The same mutation was found in 53 collection sites among 70 locations surveyed. The F1269C frequencies were lower in the middle to north part of Vietnam, but they were higher in the areas neighboring big cities such as Dong Ha, Hue, Da Nang, Tam Ky, Quang Ngai, Quy Nhon, and Nha Trang (Figure 1). Overall allelic frequency of F1269C was 21.6%. In the south of Vietnam, high frequencies of F1269C were observed at almost all collection sites except for the southern mountainous area (site nos. 3021, 3042, 3050, 3051, 5041, and 5042) located at an elevation of 500–1000 m. The allelic frequency of F1269C was 0–6.3 in these southern mountainous area even though the susceptibility indices were of maximum value (36), indicating low pyrethroid susceptibility. The highest frequency was 87.5% in site no. 1091 in Da Nang city. The percentage of homozygous of mutations was also highest in this site (75.0%). Univariate analysis was performed by categorizing two parameters into each two levels, ≥10 and <10 for the susceptibility indices and >0 and 0 for the allelic frequency or percentage of homozygous of F1269C, respectively. There was, however, no significant correlation between the susceptibility indices and the allelic frequencies (χ^2^ = 3.31, *P* = 0.069) or the percentage of homozygous (χ^2^ = 2.34, *P* = 0.126) of F1269C mutations.

**Table 1:** Susceptibility index and allelic frequency of the mutations in *Aedes aegypti* collected in used tires in Vietnam.

Collection Site1)	Longitude	Latitude	Susceptibility Index2)	Allelic frequency (AF %) and % of homozygous per larvae (RR %)					
				V1016G3)			F1269C4)		
				N	AF %	RR %	N	AF %	RR %
1035	19.57	105.81	8	12	0	0	12	4.2	0
1037	19.49	105.81	1	12	0	0	10	0	0
1038	19.45	105.78	1	12	0	0	11	0	0
1064	17.92	106.47	6	12	0	0	12	0	0
1066	17.70	106.44	3	12	0	0	12	16.7	0
1068	17.46	106.63	3	12	0	0	12	0	0
1073	17.05	107.03	10	12	0	0	12	4.2	0
1087	16.27	108.02	12	12	0	0	11	72.7	45.5
1088	16.13	108.12	24	12	0	0	12	62.5	33.3
1091	16.03	108.19	15	12	0	0	12	87.5	75.0
1098	15.41	108.68	6	10	0	0	11	36.4	9.1
1099	15.33	108.74	6	10	20.0	0	12	4.2	0
1102	14.65	109.06	5	10	0	0	6	58.3	20.0
1104	13.89	109.12	36	8	0	0	10	5.0	0
1105	13.80	109.15	18	na	na	na	7	0	0
1107	13.56	109.25	30	12	0	0	11	31.8	0
1114	12.28	109.19	30	9	0	0	9	44.4	22.2
1116	11.92	109.15	36	11	0	0	9	0	0
1119	11.61	108.99	36	12	0	0	10	30.0	10.0
1123	11.22	108.43	36	12	0	0	11	9.1	0
1130	10.96	106.96	36	12	0	0	10	35.0	0
3016	12.69	108.11	36	12	0	0	12	8.3	0
3021	12.18	108.14	36	11	0	0	11	0	0
3027	11.75	108.39	36	12	0	0	12	12.5	8.3
3033	11.59	108.95	18	10	0	0	11	0	0
3042	12.61	107.92	36	9	0	0	10	0	0
3050	11.99	107.67	36	3	0	0	8	6.3	0
3051	11.98	107.59	36	12	0	0	12	0	0
3055	11.80	107.19	36	11	0	0	12	29.2	0
3056	11.71	107.10	36	11	0	0	12	4.2	0
3058	11.76	107.01	36	3	0	0	7	0	0
3059	11.81	106.95	36	12	0	0	12	8.3	0
3061	11.55	106.90	36	9	0	0	10	35.0	0
3062	11.46	106.88	36	12	0	0	9	16.7	0
3063	11.43	106.86	36	12	0	0	10	20.0	0
3068	10.99	106.65	36	9	0	0	10	25.0	0
4019	19.38	105.74	6	10	0	0	12	0	0
4029	18.46	105.77	6	9	0	0	11	0	0
4055	16.81	107.12	12	11	0	0	12	50.0	16.7
4057	16.70	107.23	18	11	0	0	10	60.0	40.0
4062	16.52	107.48	4	na	na	na	na	na	na
4081	15.79	108.32	18	1	0	0	4	37.5	25.0
4105	13.96	109.07	12	12	0	0	12	25.0	0
4119	12.55	109.17	6	11	0	0	12	4.2	0
4121	12.40	109.18	36	11	0	0	12	8.3	0
4132	11.22	108.50	36	10	0	0	11	22.7	0
4147	10.94	107.12	12	12	0	0	11	45.5	9.1
4148	10.95	106.99	36	9	0	0	12	4.2	0
4152	10.66	106.56	24	11	0	0	10	60.0	40.0
4153	10.61	106.45	36	10	0	0	11	31.8	9.1
4158	10.41	106.07	36	12	0	0	12	45.8	25.0
4161	10.27	105.92	36	10	0	0	11	59.1	27.3
4166	9.81	105.82	12	6	0	0	11	45.5	27.3
4167	9.66	105.93	36	12	0	0	11	18.2	0
4174	9.24	105.48	36	12	0	0	12	29.2	0
4177	9.16	105.22	36	12	0	0	6	8.3	0
4181	8.94	105.01	36	12	0	0	11	27.3	0
5001	12.29	109.19	12	12	0	0	11	27.3	9.1
5003	12.40	109.18	36	12	0	0	11	9.1	0
5009	12.76	108.73	12	11	0	0	12	0	0
5030	11.61	108.99	12	12	0	0	12	8.3	0
5035	12.19	109.17	36	12	0	0	11	40.9	9.1
5041	12.31	107.45	36	13	0	0	11	0	0
5042	12.42	107.58	36	12	0	0	11	0	0
5052	11.79	107.18	36	11	0	0	9	16.7	0
5053	11.81	106.95	36	12	0	0	10	0	0
5055	11.66	106.90	36	12	0	0	11	45.8	27.3
5056	11.54	106.91	36	12	0	0	9	0	0
5062	10.99	106.65	2	11	0	0	11	22.7	0
5070	10.54	106.39	30	12	0	0	11	40.9	18.2
5076	9.66	105.93	36	12	0	0	11	36.4	9.1
5080	8.78	105.00	18	12	0	0	12	41.7	16.7
Total				756	0.26	0	757	21.6	7.4

1) Larvae were collected on 7–16 December, 2006 (1035–1130); 15–20 May, 2007 (3016–3068); 1–12 July, 2007 (4019–4181); and 7–16 January, 2008 (5001–5080).

2) Referred from Kawada et al. (2009).

3) Accession AB517741.

4) Accession AB517740.

## Discussion

The correlation between adult and larval susceptibility against pyrethroids [14],[15] seems to be common. This is, however, not always true for all cases, since mosquitoes may develop different resistance mechanisms through different metabolic pathways in the larval and adult stages. In the present study, we focused on knockdown resistance that is dependent on nervous system insensitivity controlled by the *kdr* gene, but which is not chiefly dependent on enhancement of metabolic activity [3].

The present study revealed that there seemed to be no distribution of I1011 and L1014 point mutations and very low frequency distribution of the V1016G mutation in *Ae. aegypti* collected from used tires in Vietnam. This result is in contrast to the study by Rajatileka et al. [16] in which the widespread distribution of V1016G and I1011V point mutations in the same species in Thailand were reported. Among the 756 larvae collected from used tires at 70 locations, we could find only two individuals that were heterozygous for the V1016G mutation. Therefore, this point mutation is thought to be minor in Vietnam but not negligible, since we found the same mutation in two different colonies of larvae collected from water jars in southern Vietnam (Cai Lay district, Tien Giang province and Chau Thanh district, Hau Giang province). In contrast, the distribution of the novel point mutation, F1269C, was widespread in Vietnam. Yanola et al. first found this novel mutation in segment 6, domain III, of the voltage-gated sodium channel gene in DDT/permethrin-resistant *Ae. aegypti* (PMD-R strain) [13]. They also demonstrated that the bioassay of F1 offspring arising from the cross of permethrin-susceptible, F1269-homozygous parents (PMD) and permethrin-resistant C1269-homozygous parents (PMD-R) and the subsequent backcrossing of F1 individuals to their resistant parental strain showed that C1269 segregates as a recessive allele in conferring permethrin resistance. F1269C frequencies were lower in the middle to northern part but became high in the southern part of the country. The tendencies for F1269C mutation frequencies to be higher in areas neighboring big cities may indicate that higher amounts of pyrethroids have been used in this area than in rural areas. High frequencies of F1269C mutation were observed in almost all collection sites in the southern part of Vietnam. The frequencies of F1269C mutation in the southern mountainous area (site nos. 3021, 3042, 3050, 3051, 5041, and 5042), however, were very low, indicating the possible existence of another resistance mechanism, such as another novel point mutations or development of another enhanced detoxification pathway associated with pyrethroid insensitivity. The presence or co-presence of such other resistance mechanisms might be one of the reasons that no significant correlation between the susceptibility indices and F1269C frequencies was observed in the present study. O'Reilly et al. indicated that the point mutation (F1538I, which is identical to F1273I by the notational system used in the present paper and locates in the vicinity of F1269) on the same segment (segment 6 of domain III) as F1269C has the potential importance in pyrethroid binding [17]. More attention should be paid, therefore, on the point mutations on domain III. The overall percentage of homozygous of F1269C seems to be still low (7.4%) in the present situation. However, extensive and uncontrolled frequent use of photo-stable pyrethroids might be a strong selection pressure for this mutation to cause serious problem in controlling of dengue fever in Vietnam.

Kawada et al. [3] reported that the susceptibility of *Ae. aegypti* to *d*-allethrin decreased significantly with the decrease in latitude of the collection points. Vu et al. [5] and Huber et al. [6] reported a similar tendency in pyrethroid susceptibility in the same species in Vietnam. The above authors concluded that the longer and extended use of pyrethroids for malaria control might be one of the factors causing the discrepancy in pyrethroid susceptibility in different regions in the southern areas and Central Highlands. Actually, after abandonment of DDT sprays in 1995, many photostable pyrethroids (λ-cyhalothrin, α-cypermethrin, deltamethrin, and permethrin) have been used to treat the interiors of houses as a residual spray and in the manufacture of pyrethroid-impregnated bed nets for malaria control in Vietnam [1], [18]–[20]. Additionally, 21,000 liters of photostable pyrethroid formulations, such as λ-cyhalothrin, deltamethrin, and permethrin, were reportedly used for dengue control in 20 southern provinces in 2007 [4]. The extensive use of photostable pyrethroids, therefore, seems to have been very common in southern Vietnam. Pyrethroid treatment for malaria vector control appears to have been intensively conducted in the interior and along the periphery of human habitation areas, where incidentally, the breeding and resting sites of *Ae. aegypti* are located. This may account for the strong selection pressure toward *Ae. aegypti* since *Ae. aegypti* is generally a domestic and endophagic species with a greater preference for indoor breeding [21]–[25].

The presence of *Ae. aegypti* was first recorded in Vietnam in 1915 [26], and since then, it has maintained hyperendemicity in most cities. Although *Ae. aegypti* is one of the most important disease vector mosquitoes, and many studies detailing its insecticide resistance, especially in Asian and South and Central American countries, have been reported [10], [27]–[35], few studies concerning the insecticide resistance of *Ae. aegypti* have been published in Vietnam [5],[6]. The situation is the same in the case of malaria vectors, except for the recent article by Bortel et al. [36] in which the high and widespread pyrethroid resistance in *Anopheles dirus s.l.* and *Anopheles epiroticus* in central and southern Vietnam was first reported. As the above few papers have indicated, pyrethroid resistance in these vector mosquitoes seems to be high. Pyrethroids provide one of the most promising countermeasures for controlling malaria, dengue hemorrhagic fever (DHF), and other arthropod-borne diseases. Pyrethroid resistance, therefore, will be a major problem for vector control programs, since, at present, there are no suitable chemical substitutes for pyrethroids. Vu et al. reported the lack of OP (organophosphate) resistance in *Ae. aegypti* colonies collected in southern Vietnam that showed high resistance to pyrethroids [5]. Therefore, the use of OP as a substitute for pyrethroids for emergence control of *Ae. aegypti* in the epidemic area might be effective temporarily. However, long term and extended use of OP may eventually result in the same resistance problem as that seen with pyrethroids. A regular monitoring system for insecticide susceptibility, including a simple biochemical evaluation system that can elucidate the modes of resistance at the mosquito population level, should be a priority. Additionally, development of new chemicals with novel modes of action that can be substituted for conventional insecticides, as well as transitional life-prolonging measures for conventional insecticides such as pyrethroids belonging to photo-unstable knockdown agent groups [37],[38], rotational use of plural insecticides with different mode of actions, and basic biochemical and genetic research to support the above strategies, are essential for the effective management of insecticide resistance.
